# Supplementary figures and images for: Curcumin induces mitochondrial dysfunction-associated oxidative DNA damage in ovarian cancer cells
Source: PLoS One. 2025 Mar 31;20(3):e0319846. doi: 10.1371/journal.pone.0319846 (PMC11957317; doi:10.1371/journal.pone.0319846)

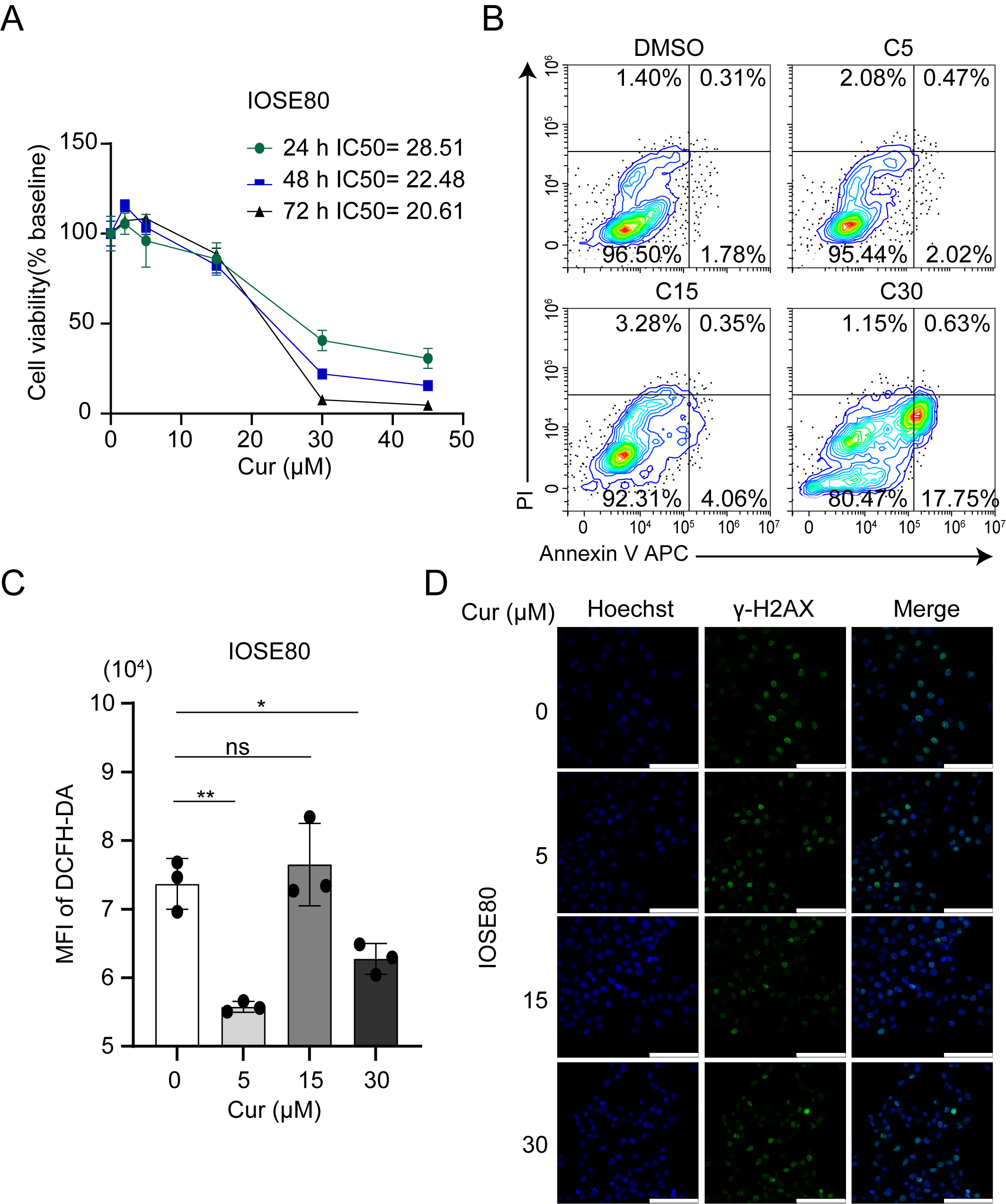

Supplement: S1 Fig — (A) IOSE80 cells were exposed to curcumin (2-45 μM) or vehicle control (0.1% DMSO) for 24 h, 48 h and 72 h. Cell viability was measured by CCK-8 assay. The experiments were performed in quadruplicate. (B) IOSE80 cells were exposed to 5, 15 and 30 μM curcumin for 48 h. 0.1% DMSO was used as control. * p < 0.05, **p < 0.01, ***p < 0.001, ****p < 0.0001, when compared with control group. ROS generation was measured using oxidation-sensitive fluorescent probe (DHE) by flow cytometry. Means and S.D.s of three repeats were shown. (C) IOSE80 cells were exposed to 5, 15 and 30 μM curcumin for 48 h. 0.1% DMSO was used as control. Cells were processed by flow cytometry using Annexin V and PI staining. Results shown are representative of three independent experiments. (D) IOSE80 cells were exposed to 5, 15 and 30μM curcumin for 12 h. 0.1% DMSO was used as control. IF images showing the γ-H2AX; Hoechst was used for the nuclear staining. Scale bar, 100 μm. (TIF) [file pone.0319846.s001.tif]

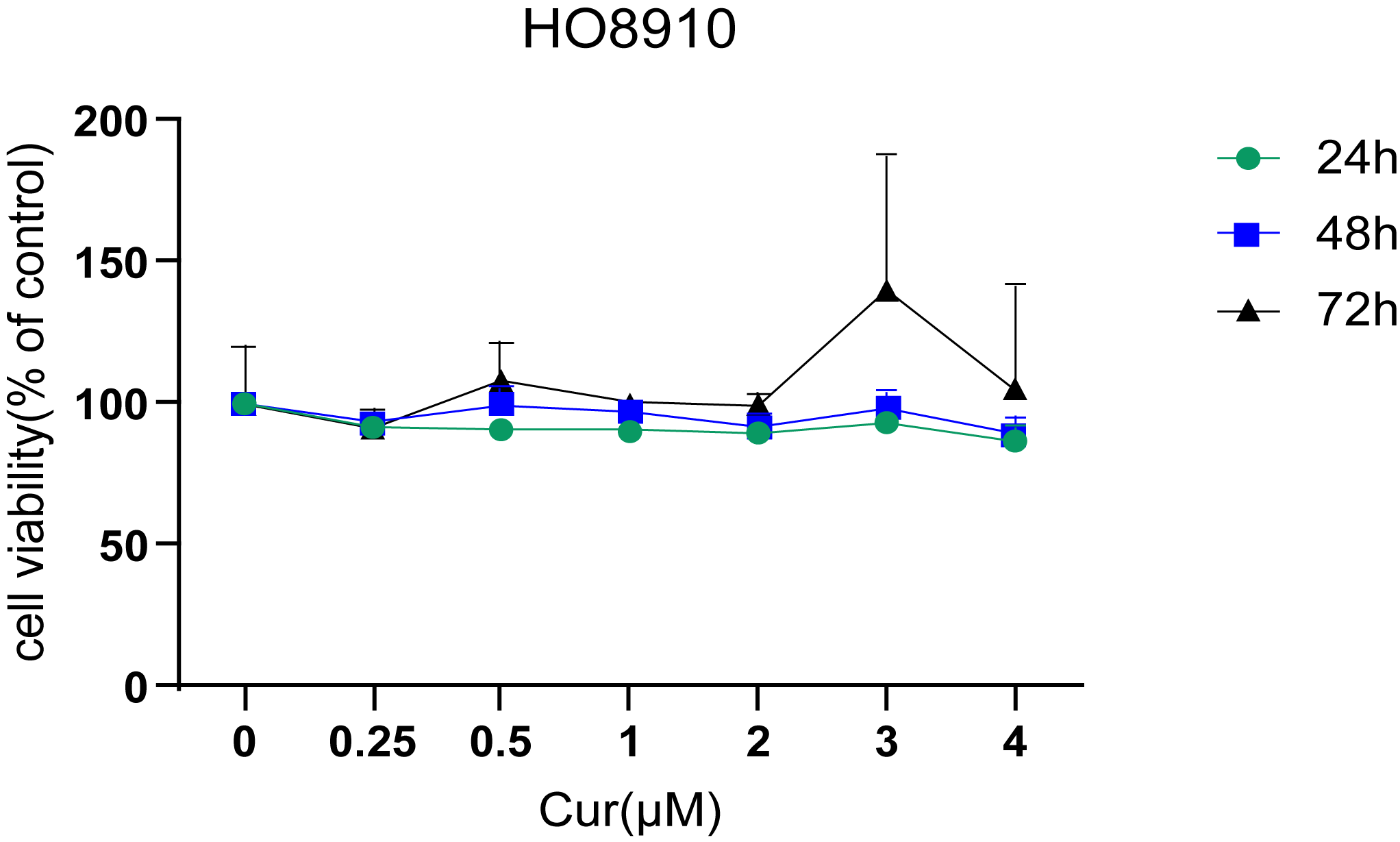

Supplement: S2 Fig — HO8910 cells were exposed to curcumin (0.25-4 μM) or vehicle control (0.1% DMSO) for 24 h, 48 h and 72h. Cell viability was measured by CCK-8 assay. The experiments were performed in quadruplicate. (TIF) [file pone.0319846.s002.tif]
