# Supplementary material for: Curcumin induces mitochondrial dysfunction-associated oxidative DNA damage in ovarian cancer cells
Source: PLoS One. 2025 Mar 31;20(3):e0319846. doi: 10.1371/journal.pone.0319846 (PMC11957317; doi:10.1371/journal.pone.0319846)
Supplement: S1 File — (PDF) [file pone.0319846.s003.pdf]

Fig. 1E Human ovarian cancer cell lines HO8910, OVCAR3 wrereexposed to 0, 5, 15, 30 μM concentrations of curcumin for 48h. The expression levels of CDC25A and Cyclin B1 which are known to regulate the G2/M phase arrest, such as dose-dependent reduction of cyclin B1 and CDC25A.

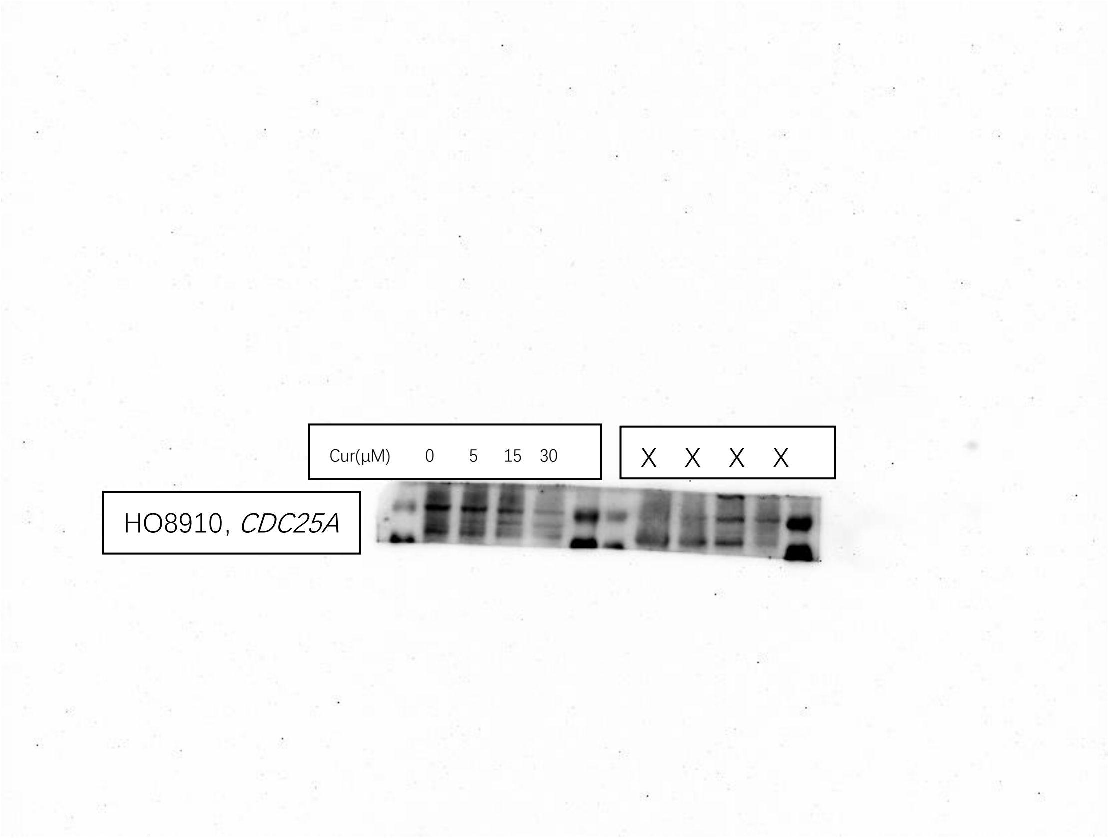

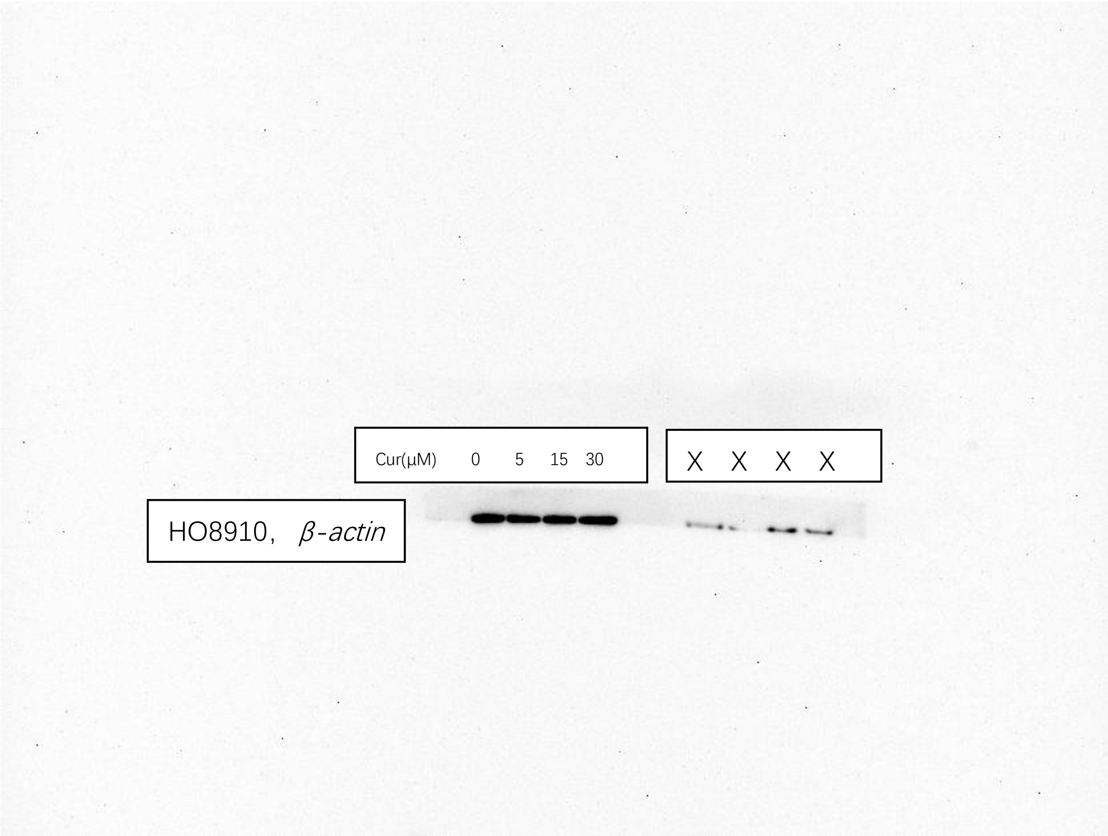
Cur(μM) 0 5 15 30
X X X X
HO8910, β-actin

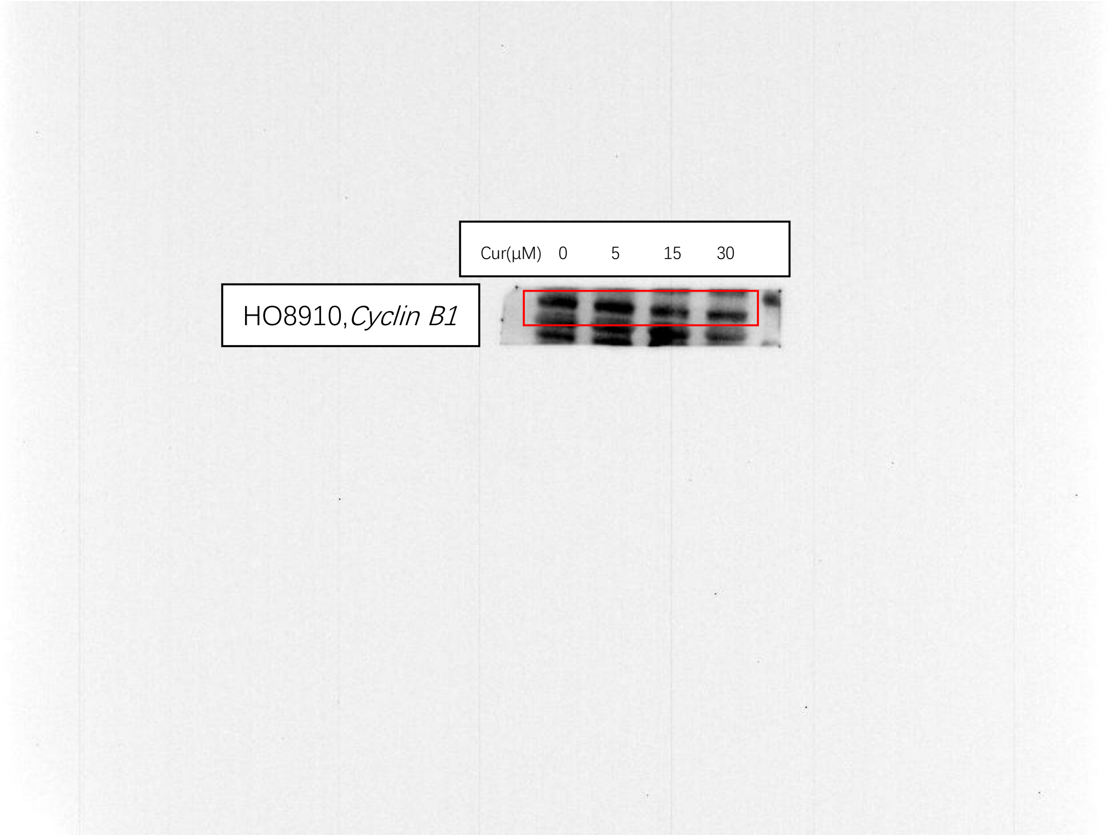
Cur(μM) 0 5 15 30
HO8910,Cyclin B1

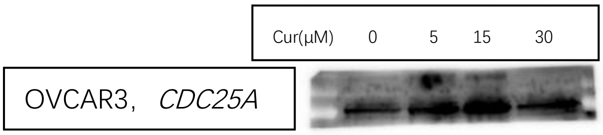
Cur(μM) 0 5 15 30
OVCAR3，*CDC25A*

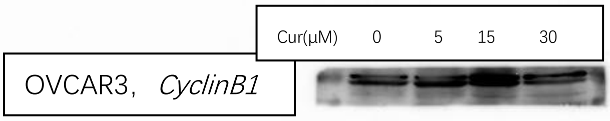
Cur(μM) 0 5 15 30
OVCAR3，*CyclinB1*

Cur(μM) 0 5 15 30

OVCAR3, *β-actin*

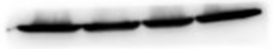

Fig.2C Human ovarian cancer cell lines HO8910, OVCAR3 were exposed to 0, 5, 15, 30 μM concentrations of curcumin for 48h. The expression levels of pro-apoptotic protein Bax and the anti-apoptotic protein Bcl2 was observed.

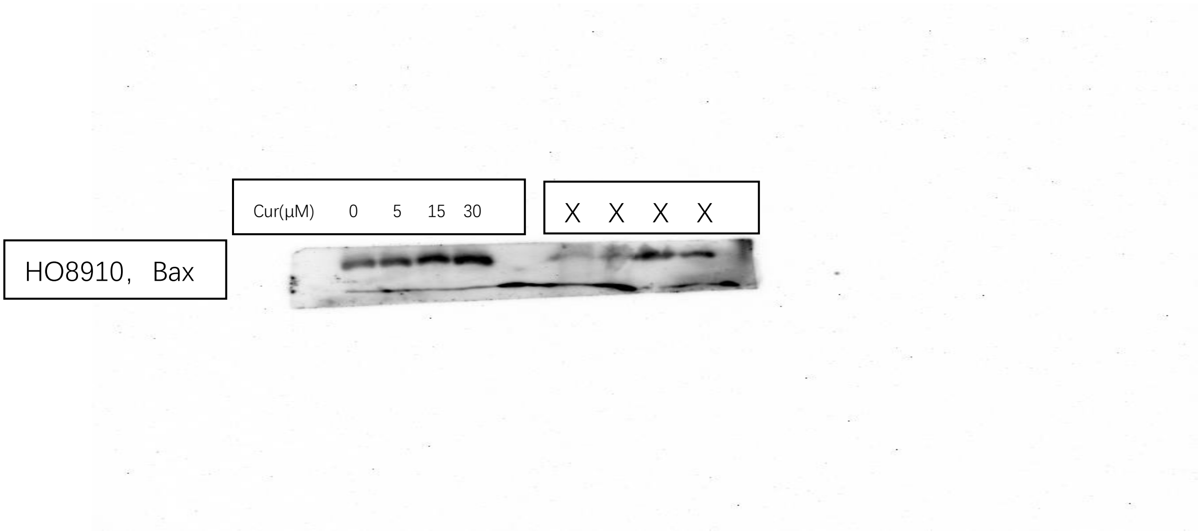

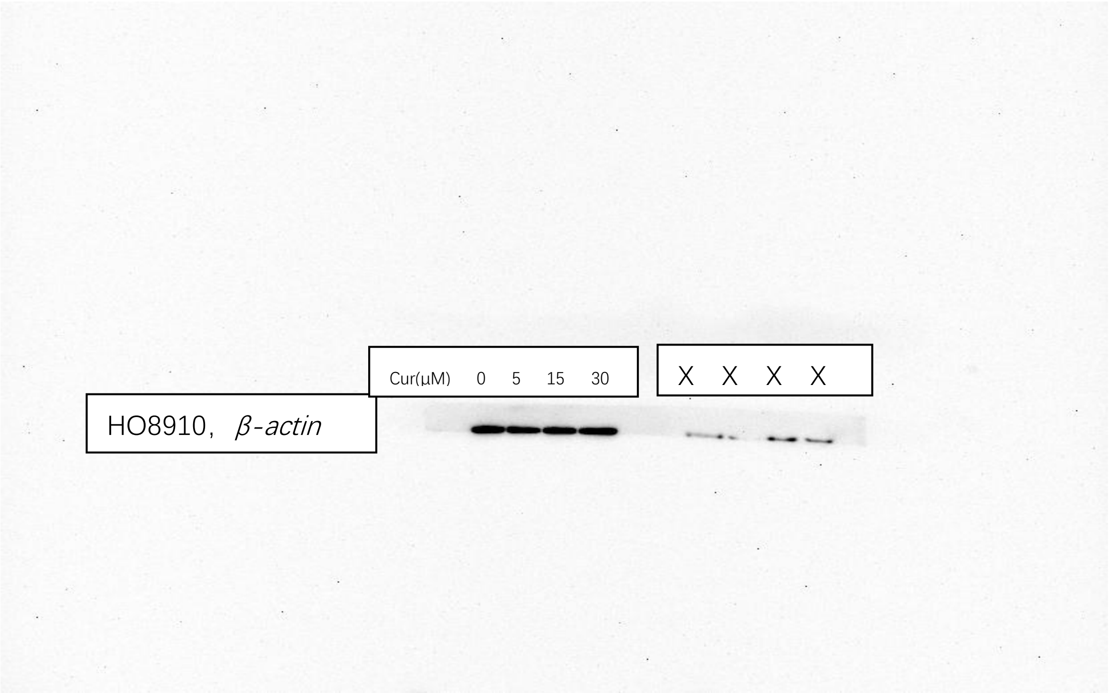

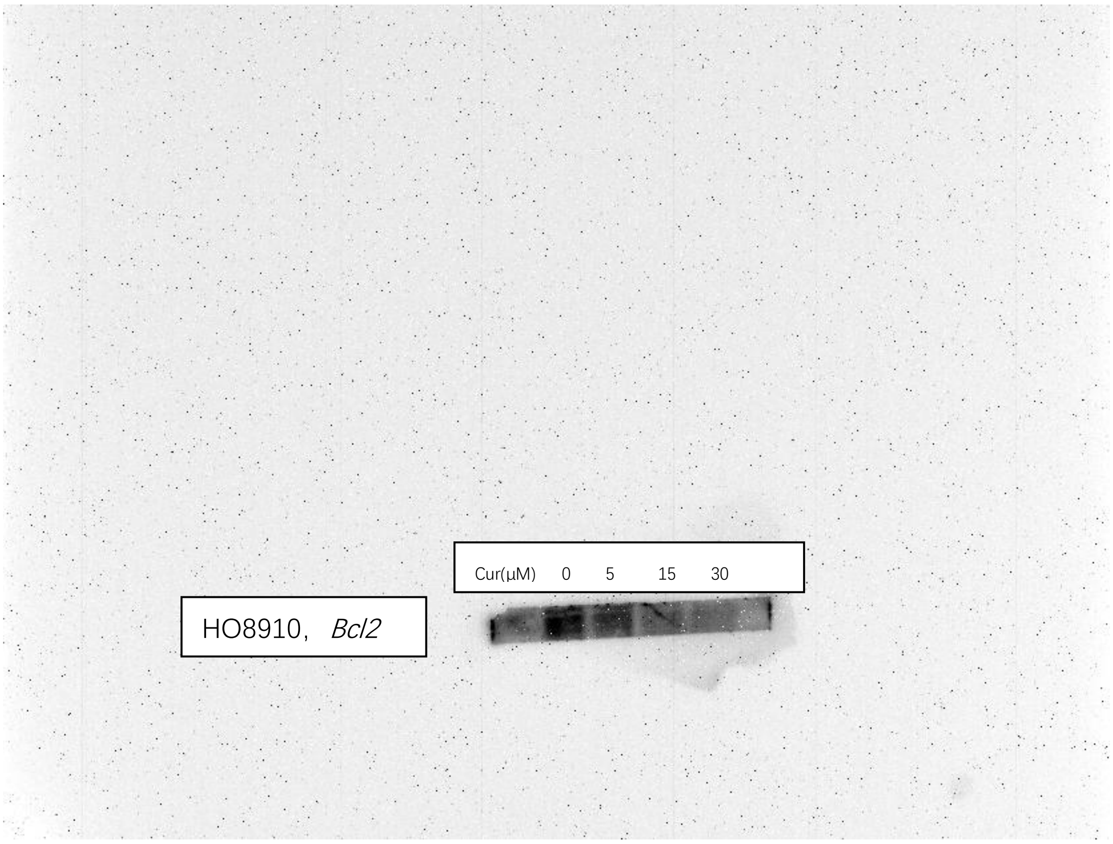
Cur(μM) 0 5 15 30
HO8910, *Bcl2*

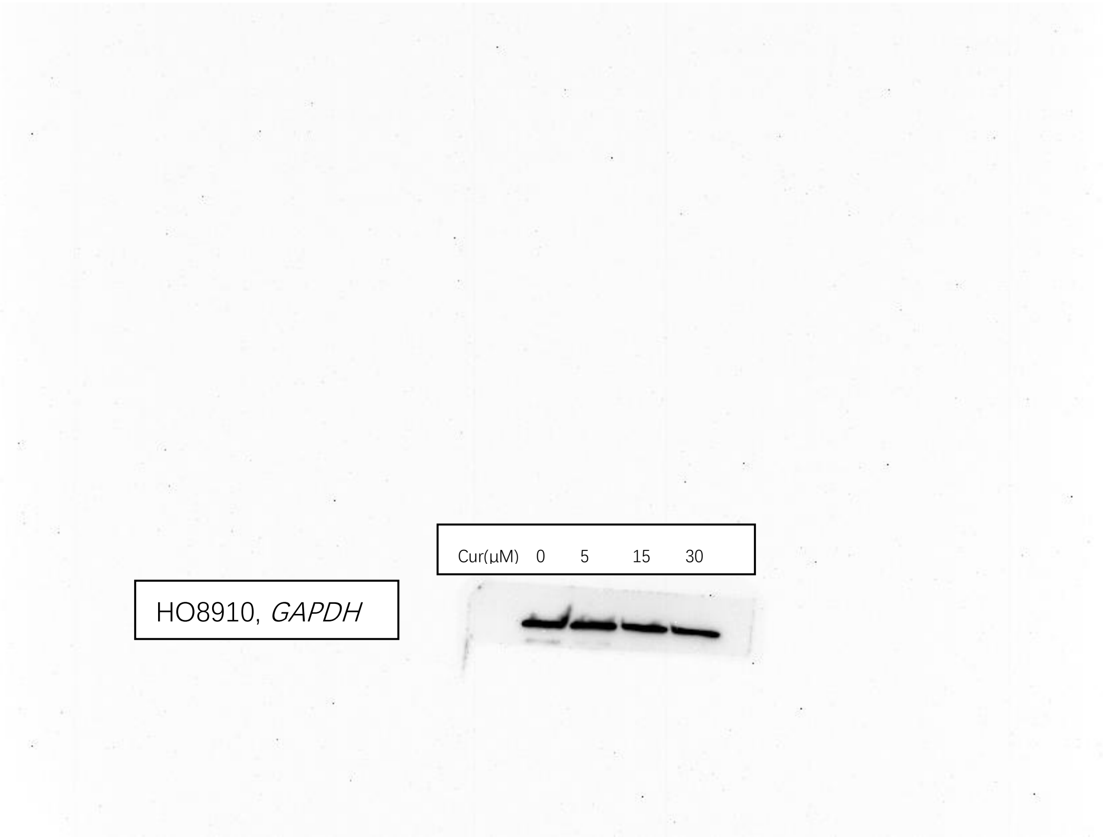
Cur(μM) 0 5 15 30
HO8910, *GAPDH*

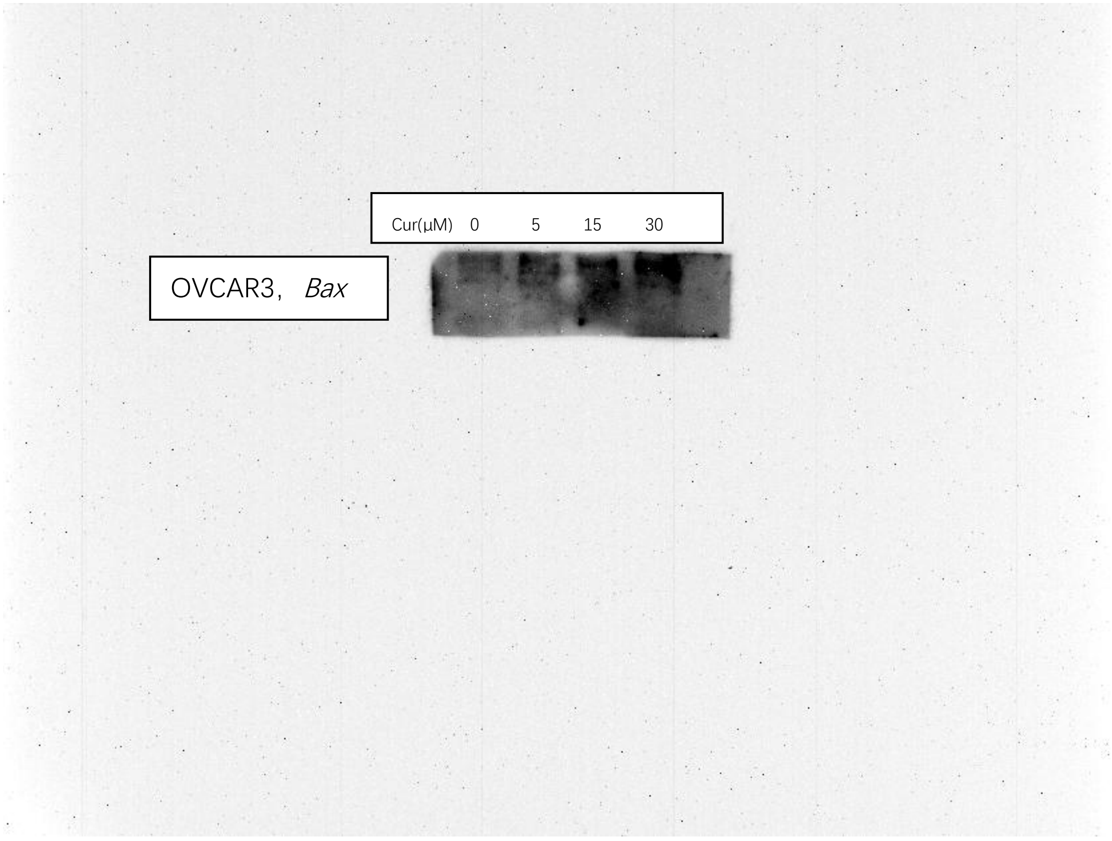
Cur(μM) 0 5 15 30
OVCAR3, *Bax*

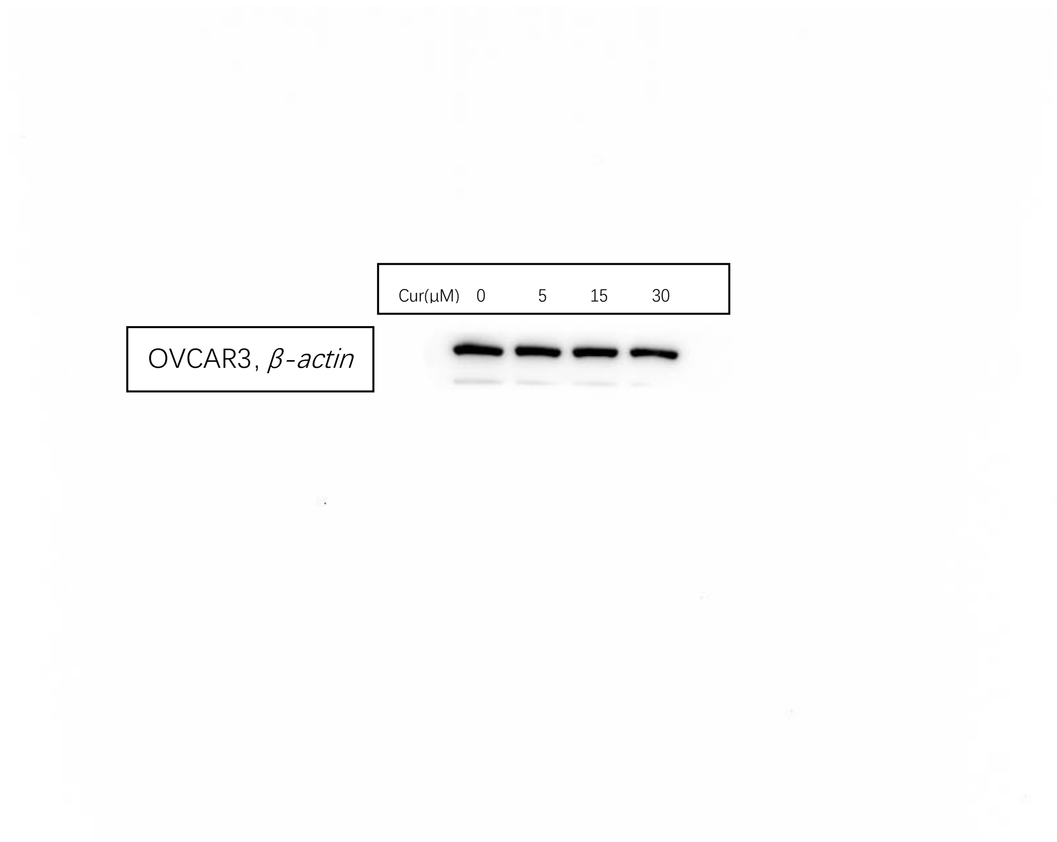
Cur(μM) 0 5 15 30
OVCAR3, *β-actin*

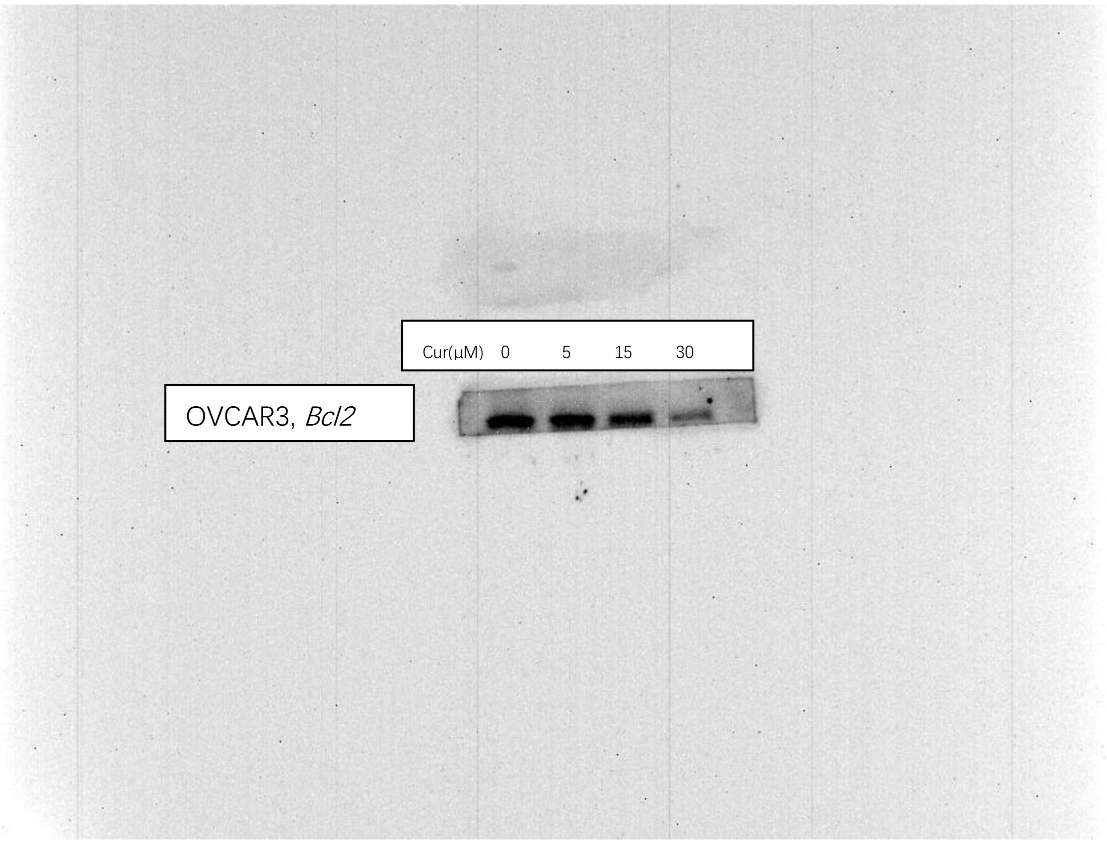
Cur(μM) 0 5 15 30
OVCAR3, *Bcl2*

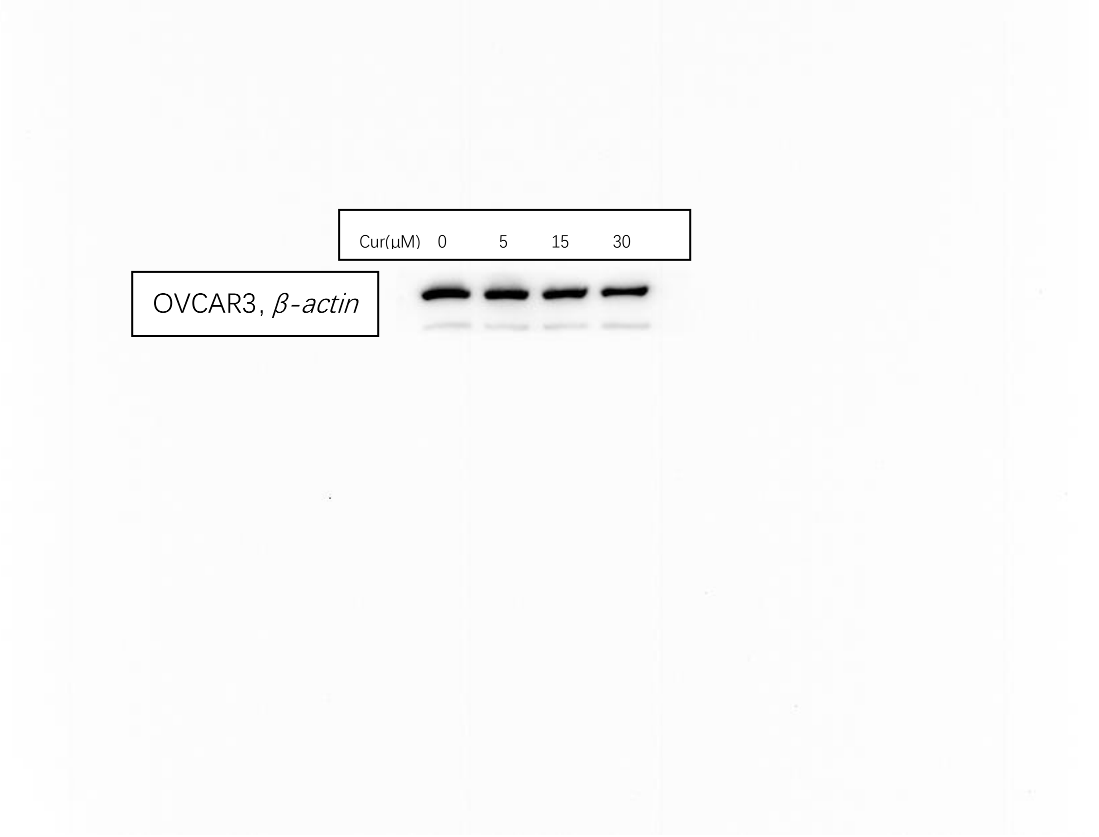
Cur(μM) 0 5 15 30
OVCAR3, *β-actin*

Fig.4G Human ovarian cancer cell lines HO8910, OVCAR3 wrereexposed to 0, 5, 15, 30 μM concentrations of curcumin for 48h.PINK1 is primarily situated in the mitochondrial inner membrane and is essential for eliminating damaged mitochondria through mitophagy.

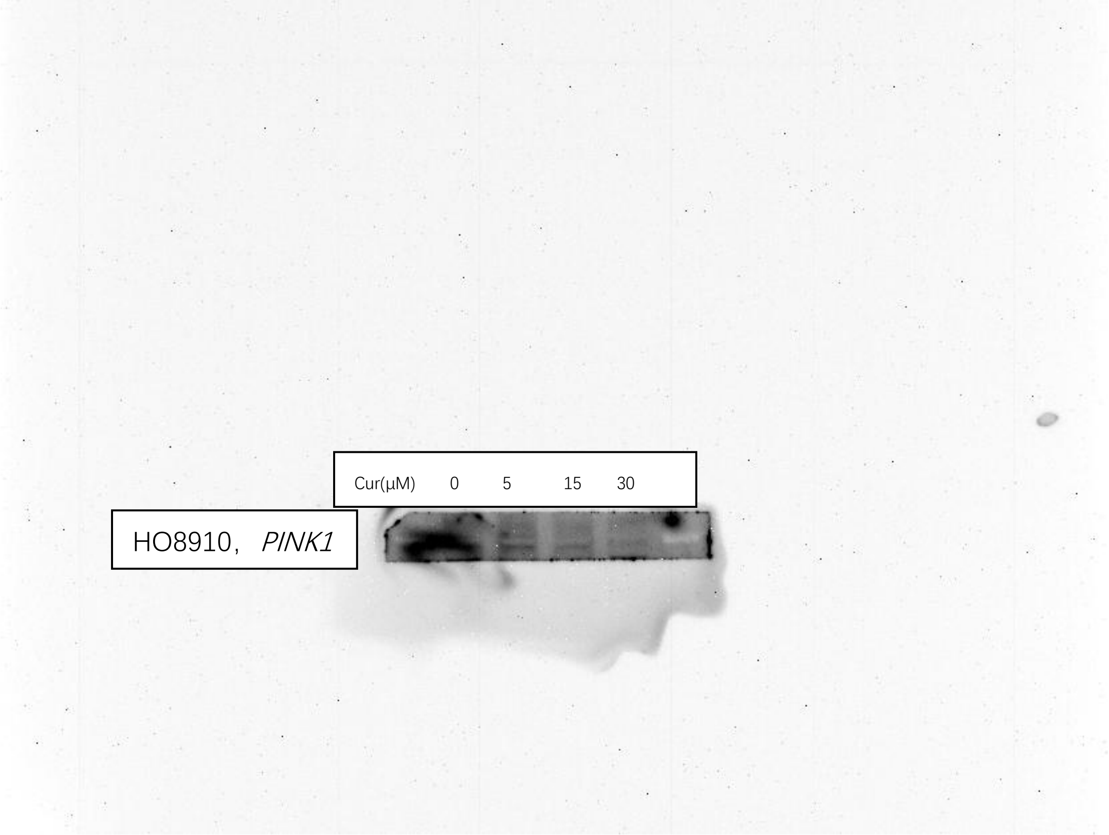

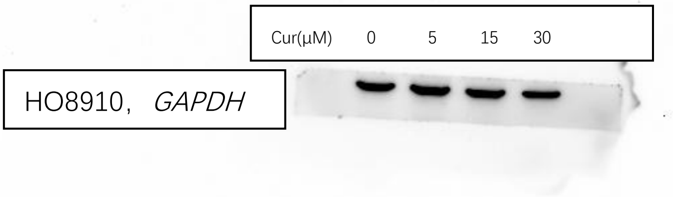
Cur(μM) 0 5 15 30
HO8910， *GAPDH*

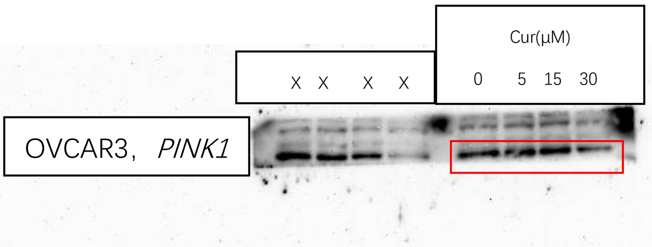
X X X X
Cur(μM)
0 5 15 30
OVCAR3， *PINK1*

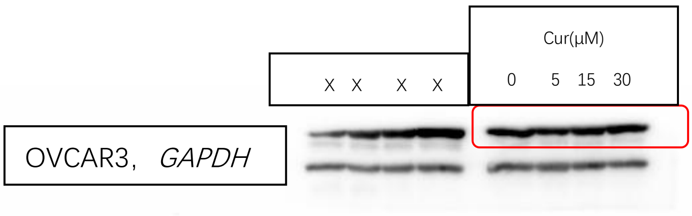
Cur(μM)
0 5 15 30
X X X X
OVCAR3， GAPDH
