# Supplementary material for: Curcumin induces mitochondrial dysfunction-associated oxidative DNA damage in ovarian cancer cells
Source: PLoS One. 2025 Mar 31;20(3):e0319846. doi: 10.1371/journal.pone.0319846 (PMC11957317; doi:10.1371/journal.pone.0319846)
Supplement: Attachment — Submitted filename: Response to Reviewers.doc [file pone.0319846.s004.doc]

Responses to reviews (original comments by reviews are in blue color)

**Reviewer # 1:**

1.Comment: Issue: The study investigates curcumin's effect on ovarian cancer cells, which is a well-explored area. Many studies have already demonstrated curcumin’s anticancer properties, particularly its role in inducing oxidative stress, apoptosis, and mitochondrial dysfunction. The manuscript doesn't clearly state what sets this research apart from existing studies or what unique contributions it makes to the field.

1.Reply: Thank you so much for your comments on our manuscript. The anticancer properties of curcumin, especially its role in inducing oxidative stress, apoptosis and mitochondrial dysfunction, have indeed been demonstrated in many studies. However, this effect of curcumin on ovarian cancer cells has not been well characterized. Previous studies tend to concentrate on other aspects of the cellular response to curcumin, as shown below:

Ravindran F, Mhatre A, Koroth J, Narayan S, Choudhary B. Curcumin modulates cell type-specific miRNA networks to induce cytotoxicity in ovarian cancer cells. Life Sci. 2023 Dec 1;334:122224. doi: 10.1016/j.lfs.2023.122224. Epub 2023 Oct 30. PMID: 38084671.

Liu Y, Shen Z, Zhu T, Lu W, Fu Y. Curcumin enhances the anti-cancer efficacy of paclitaxel in ovarian cancer by regulating the miR-9-5p/BRCA1 axis. Front Pharmacol. 2023 Jan 10;13:1014933. doi: 10.3389/fphar.2022.1014933. PMID: 36703740; PMCID: PMC9871306.

Zheng N, Liu S, Zeng H, Zhao H, Jin L. Molecular Mechanism of Curcumin Derivative on YAP Pathway against Ovarian Cancer. J Clin Med. 2022 Dec 5;11(23):7220. doi: 10.3390/jcm11237220. PMID: 36498794; PMCID: PMC9740724. Our study shows that curcumin treatment of ovarian cancer cells induces mitochondrial dysfunction-associated elevated ROS and DNA damage, ultimately leading to their apoptotic fate.

2. Comment: Abstract: The abstract is concise and covers key aspects of the study. However, it could be enhanced by explicitly stating the novelty or unique findings of the research. For instance, mentioning more specific implications for clinical treatment could provide clarity.

Consider adding brief details about the doses used and results from in vivo models for a more complete overview.

2.Reply: We cited literature “ Tate, J.G., et al., COSMIC: the Catalogue Of Somatic Mutations In Cancer. Nucleic Acids Res, 2019. 47(D1): p. D941-D947.” as reference [2]. We added “This effect of curcumin is independent of the BRCA mutation status” in Abstract. We also added “ According to the Catalogue Of Somatic Mutations In Cancer (COSMIC), ovarian cancer cell lines such as OVCAR3, A2780, SKOV3, HEY, etc. are free of BRCA mutation, and only HO8910 carries BRCA1 5382C mutation. Therefore, the HO8910 cell line and the OVCAR3 cell line were selected for this study” on page 3. Consider adding brief details about the doses used and results from in vivo models for a more complete overview. We added “ 200 mg/kg intraperitoneal infusion daily ” and “ oxidative damage, and mitochondrial accumulation ” on page 2. We added: and can be potentially used in combination with other DNA repair-interfering therapeutics, such as PARP inhibitor, in the treatment of ovarian cancer at the end of the abstract.

3.Comment: Introduction: While the rationale for using curcumin is well presented, it might benefit from more information about the gap in current literature that the study aims to address. The introduction could be improved by stating the specific hypotheses or questions being tested more explicitly.

3.Reply: We added “While an anticancer effect of curcumin was also reported on ovarian cancer, whether or not curcumin induces mitochondrial dysfunction has not been clarified , and”. they were supplemented by citations to the relevant literature on page 4.

4.Comment: The methods section does not explicitly mention how the different concentrations of curcumin (2 to 45 μM) were selected. Typically, drug concentrations like curcumin are chosen based on previous studies and determined by the IC50 or effective dose ranges in laboratory experiments. It is likely that the researchers selected these concentrations based on prior research findings or pilot studies to assess dose-dependent effects. To provide a more precise explanation in the manuscript, it would be helpful for the authors to clarify how these concentrations were chosen, for example, based on previous studies or preliminary experiments to determine the IC50. This information could contribute to a better understanding of the results.

4.Reply: We selected the different concentrations of curcumin (2 to 45 μM) were based on previous studies or preliminary experiments. At first, we used a low concentration of curcumin and found that curcumin had no effect on the viability of ovarian cancer cells below a concentration of 5 μM, so the curcumin concentration was increased to more than 5 μM for the IC 50 assay, as shown in figure below ( and we submitted this result as Figure S2).

5. Comment: the methods section could benefit from more specific details on the statistical methods used to ensure reproducibility. Including the number of technical and biological replicates for each experiment (e.g., for flow cytometry) would improve transparency.

5.Reply: We added “ The experiment was repeated 3 times and each contained at leasthree biological replicates.” on page 6. The other experiments e.g. EdU proliferation assay, we discribed flow cytometry as “Ratio of EdU positive cells is shown as the mean ± S.D. from three independent experiments.” And we added “ The experiment was repeated 3 times and each contained at leasthree biological replicates.” on page 6, 7, 8, 9, 10 “Material and method ”.

6.Comment: When using ANOVA to compare more than two groups, post-hoc tests (e.g., Tukey’s test or Bonferroni correction) are necessary to identify specific group differences. The manuscript does not mention whether such post-hoc tests were performed. Omitting this step can result in incomplete analysis when significant differences are detected by ANOVA.

6.Reply:Tukey’s test was used to determine the statistical significance between experimental groups. So we added “and Tukey’s test were was” on page 10.

7.Comment: While the results are comprehensive, there is a lack of clear narrative flow between the experiments. For instance, the connection between the mitochondrial dysfunction in vitro and tumor suppression in vivo isn’t fully explained. Some results, like the decreased ROS levels in non-cancerous cells, are intriguing but aren’t discussed adequately.

Comparison: Well-organized manuscripts have a clear logical flow from one result to the next, and each finding is discussed in sufficient depth. A stronger explanation of the significance of each result and its connection to the overall hypothesis would enhance the manuscript’s coherence.

7.Reply: Thank you for your wise suggestions! To well-organized one result to the next, we added “During cell culture, we found that curcumin-treated ovarian cancer cells tended to float and the number of floating cells increased with increasing curcumin concentration.” on page 12. “The aforementioned experiments revealed that curcumin inhibits the proliferation and induces apoptosis of ovarian cancer cells by up-regulating oxidative stress, and to substantiate this notion, we attempted a rescue experiment with the antioxidant NAC” on page 14, 15. “We further explored whether the tumour-suppressive effects and mechanisms of curcumin in in vivo experiments were consistent with those in vitro.” on page 15.

Some literature revealed that curcumin can inhibit ROS levels and oxidative stress in non-cancerous cells, e.g. “Curcumin has been reported to possess antioxidant effect in many other pathophysiological settings. For example, it inhibits oxidative stress in hepatocytes through activation of PPAR-α[31]. Curcumin ameliorates aortic endothelial injury by reducing ROS [32]. It also ameliorates endothelial damage in rat retina by inhibiting ROS production[33]. Curcumin was shown to reduce ROS levels, mainly by restoring NO levels, inhibiting endothelial inflammation and oxidative damage, and reducing leukocyte adhesion and endothelial cell apoptosis[34]. Consistently, ROS level and DNA damage were not elevated in ovarian epithelial cells. Thus, the oxidative stress-inducing effect of curcumin is probably specific to cancer cells.” in our study is consistent with the results of the previous study on page 18, 19.

8.Comment: The manuscript does not adequately address the study’s limitations, such as the relevance of in vitro findings to clinical settings or the potential off-target effects of curcumin. A lack of critical reflection can suggest a biased interpretation of the results.

8.Reply: Considering the limitations of this study and the off-target effects of curcumin. We added “To date, more than 230 clinical trials have been investigated to understand the pharmacological aspects of curcumin in the human system[43]. Although curcumin supplements are one of the best-selling botanicals with encouraging preclinical results, questions remain about bioactivity in human body [44].Curcumin has low solubility in water, is unstable under physiological pH conditions and is rapidly metabolised, which leads to its low oral bioavailability and poor blood-brain barrier permeability[45]. Due to the problems associated with the bioavailability of curcumin and off-target effects such as nausea and diarrhea, the application of nanotechnology to improve bioavailability and mitigate side effects could be further explored and developed.” And they were supplemented by citations to the relevant literature on page 19, 20.

**Reviewer # 2:**

1.Comment: The research examines the impact of curcumin on ovarian cancer cells, a topic that has been extensively studied. Numerous investigations have previously validated curcumin's anticancer effects, especially regarding its ability to promote oxidative stress, induce apoptosis, and cause mitochondrial dysfunction. However, the manuscript fails to clearly articulate how this study distinguishes itself from previous research or what novel insights it offers to the field.

1.Reply: 1.Reply: Thank you so much for your comments on our manuscript. The anticancer properties of curcumin, especially its role in inducing oxidative stress, apoptosis and mitochondrial dysfunction, have indeed been demonstrated in many studies. However, this effect of curcumin on ovarian cancer cells has not been well characterized. Previous studies tend to concentrate on other aspects of the cellular response to curcumin, as shown below:

Ravindran F, Mhatre A, Koroth J, Narayan S, Choudhary B. Curcumin modulates cell type-specific miRNA networks to induce cytotoxicity in ovarian cancer cells. Life Sci. 2023 Dec 1;334:122224. doi: 10.1016/j.lfs.2023.122224. Epub 2023 Oct 30. PMID: 38084671.

Liu Y, Shen Z, Zhu T, Lu W, Fu Y. Curcumin enhances the anti-cancer efficacy of paclitaxel in ovarian cancer by regulating the miR-9-5p/BRCA1 axis. Front Pharmacol. 2023 Jan 10;13:1014933. doi: 10.3389/fphar.2022.1014933. PMID: 36703740; PMCID: PMC9871306.

Zheng N, Liu S, Zeng H, Zhao H, Jin L. Molecular Mechanism of Curcumin Derivative on YAP Pathway against Ovarian Cancer. J Clin Med. 2022 Dec 5;11(23):7220. doi: 10.3390/jcm11237220. PMID: 36498794; PMCID: PMC9740724. Our study proposes that curcumin treatment of ovarian cancer cells induces mitochondrial dysfunction-associated elevated ROS and DNA damage, ultimately leading to their apoptotic fate.

2.Comment: The abstract effectively summarizes the primary components of the study; however, it would benefit from a clearer emphasis on the unique contributions of the research.

2.Reply: We cited literature “ Tate, J.G., et al., COSMIC: the Catalogue Of Somatic Mutations In Cancer. Nucleic Acids Res, 2019. 47(D1): p. D941-D947.” as reference [2]. We added This effect of curcumin is independent of the BRCA mutation status in Abstract. We also added “ According to the Catalogue Of Somatic Mutations In Cancer (COSMIC), ovarian cancer cell lines such as OVCAR3, A2780, SKOV3, HEY, etc. are free of BRCA mutation, and only HO8910 carries BRCA1 5382C mutation. Therefore, the HO8910 cell line and the OVCAR3 cell line were selected for this study” on page 3. Consider adding brief details about the doses used and results from in vivo models for a more complete overview. We added “ 200 mg/kg intraperitoneal infusion daily ” and “ oxidative damage, and mitochondrial accumulation ” on page 2. We added: and can be potentially used in combination with other DNA repair-interfering therapeutics, such as PARP inhibitor, in the treatment of ovarian cancer at the end of the abstract.

3.Comment: The introduction lays a solid foundation for the rationale behind curcumin's use. Methods: The methods section lacks clarity regarding the rationale for selecting the curcumin concentrations (ranging from 2 to 45 μM).

It would be beneficial for the authors to explain whether these concentrations were derived from previous studies or preliminary experiments.

3.Reply: We selected the different concentrations of curcumin (2 to 45 μM) were based on previous studies or preliminary experiments. At first, we used a low concentration of curcumin and found that curcumin had no effect on the viability of ovarian cancer cells below a concentration of 5 μM, so the curcumin concentration was increased to more than 5 μM for the IC 50 assay, as shown in figure below ( and we presented this result as Figure S2).

3.Comment: Furthermore, the section could improve by detailing the statistical analyses employed, including the number of biological and technical replicates for each experiment, such as those conducted via flow cytometry.

4.Reply: We added “Three technical and biological replicates per experiment at least. ” and “and Tukey’s test were” on Page 10.

5.Comment: Results: Although the results section is thorough, it lacks a cohesive narrative that connects the various experiments.

5.Reply: Thank you for your wise suggestions! To well-organized one result to the next, we added “During cell culture, we found that curcumin-treated ovarian cancer cells tended to float and the number of floating cells increased with increasing curcumin concentration.” on page 12. “The aforementioned experiments revealed that curcumin inhibits the proliferation and induces apoptosis of ovarian cancer cells by up-regulating oxidative stress, and to substantiate this notion, we attempted a rescue experiment with the antioxidant NAC” on page 14, 15. “We further explored whether the tumour-suppressive effects and mechanisms of curcumin in in vivo experiments were consistent with those in vitro.” on page 15.

6.Comment: Some findings, such as the reduction of ROS levels in non-cancerous cells, are compelling but warrant further discussion to contextualize their significance.

6.Reply: Some literature revealed that curcumin can inhibit ROS levels and oxidative stress in non-cancerous cells, e.g. “Curcumin has been reported to possess antioxidant effect in many other pathophysiological settings. For example, it inhibits oxidative stress in hepatocytes through activation of PPAR-α[31]. Curcumin ameliorates aortic endothelial injury by reducing ROS [32]. It also ameliorates endothelial damage in rat retina by inhibiting ROS production[33]. Curcumin was shown to reduce ROS levels, mainly by restoring NO levels, inhibiting endothelial inflammation and oxidative damage, and reducing leukocyte adhesion and endothelial cell apoptosis[34]. Consistently, ROS level and DNA damage were not elevated in ovarian epithelial cells. Thus, the oxidative stress-inducing effect of curcumin is probably specific to cancer cells.” in our study is consistent with the results of the previous study on page 18, 19.

We are grateful for the invaluable feedback provided by the reviewers, and we look forward to the opportunity to further improve our manuscript.

With kind regards,

Weipei Zhu, PhD
