# Supplementary material for: Curcumin induces mitochondrial dysfunction-associated oxidative DNA damage in ovarian cancer cells
Source: PLoS One. 2025 Mar 31;20(3):e0319846. doi: 10.1371/journal.pone.0319846 (PMC11957317; doi:10.1371/journal.pone.0319846)
Supplement: Attachment — Submitted filename: Response_to_Reviewers_auresp_2.doc [file pone.0319846.s005.doc]

Responses to reviews (original comments by reviews are in blue color)

**Reviewer # 3:**

The authors have performed a comprehensive analysis and efficiently conveyed their findings. Nevertheless, there are certain inquiries and recommendations that, if taken into consideration, could improve the overall caliber of the manuscript. Below are some comments about this study:

Abstract

The abstract summarizes the study’s structure, methods, and main results.

Introduction

1- Comment: First add some other examples of the beneficial effects of curcumin in reproductive system. Then follow with specifically about its role in reducing oxidative stress in female reproductive tissues under similr toxicological conditions. Use the below studies to complete these parts:

Protective effects of curcumin on chromatin quality, sperm parameters, and apoptosis following testicular torsion-detorsion in mice

Curcumin mitigates acrylamide‐induced ovarian antioxidant disruption and apoptosis in female Balb/c mice: A comprehensive study on gene and protein expressions

Curcumin and Its Nanoformulations: Exploring Therapeutic Potential in Female Reproductive Health

Reply: Thank you so much for your comments on our manuscript. We added “Curcumin has many benefits for the reproductive system, it has excellent prospects for the treatment of fertility disorders. Curcumin compensates for the deleterious effects of testicular ischaemia and improves sperm chromatin quality in mice[10], mitigates acrylamide-induced ovarian antioxidant disruption and apoptosis in female Balb/c mice[11]” in Background on page 4.

2- Comment: While the rationale for using curcumin is well presented, it might benefit from more information about the gap in current literature that the study aims to address. The introduction could be improved by stating the specific hypotheses or questions being tested more explicitly.

2. Reply: In order to further present the aims of the use of curcumin, we added “Curcumin is a commonly used gynaecological drug globally[5-7].” on page 3.

3- Comment: Please delete the last paragraph in the introduction “In this study we observed elevated oxidative stress, increased DNA damage, elevated mitochondrial reactive oxygen species (ROS) levels, decreased ATP production, increased mitochondrial number, and decreased mitochondrial autophagy in curcumin-treated ovarian cancer cells, indicating that curcumin might exert its anti-tumor effect via inducing mitochondrial stress”, this is the results of your study and it is better to add in the result or discussion.

3. Reply: We deleted the last paragraph in the introduction “In this study we observed elevated oxidative stress, increased DNA damage, elevated mitochondrial reactive oxygen species (ROS) levels, decreased ATP production, increased mitochondrial number, and decreased mitochondrial autophagy in curcumin-treated ovarian cancer cells, indicating that curcumin might exert its anti-tumor effect via inducing mitochondrial stress”, added it in the discussion, and delete previous portion “Our study revealed that curcumin treatment induced oxidative stress and DNA damage, mitochondrial dysfunction to exert its anti-cancer effects.”

4-Comment: The introduction is clear and well-organized but. It is suggested to write the objective of the study as below: whether or not curcumin induces mitochondrial dysfunction has not been defined [10, 13, 14], and the exact processes underpinning curcumin-induced oxidative stress and DNA damage are still to be thoroughly characterized. Our research may provide new insights into how curcumin may exert its anti-tumor effect by inducing mitochondrial stress.

4. Reply: We rewrote the objective of the study as you suggested “whether or not this anticancer property of curcumin is exerted through inducing mitochondrial dysfunction has not been well defined [15, 18, 19], and the exact underpinnings of curcumin-induced oxidative stress and DNA damage are still to be thoroughly characterized. Our research may help clarify whether curcumin exerts its anti-tumor effect by inducing mitochondrial stress.” on page 4.

5-Materials and Methods

It is written in detail and completely.

“Curcumin treatment of cultured cells Curcumin (S1848, Selleck Chemicals) was dissolved in DMSO at a stock con centration of 50 mM and applied to cells in culture at different final concentrations.”

Would you please clarify which cell concentration you used?

5. Reply: We descibed cell concentration “Cells then were seeded in 6-well plates at a density of 0.1×10^6^ cells/ml in complete DMEM medium” in Cell lines and Reagents, and “cell lines (1× 10^3^) were seeded in a 96-well culture plate” in Cell viability assay. We added “Single-cell suspensions were generated for each cell line and 1×10^3^ of cells were seeded into six-well tissue culture plates. Then, cells were exposed to different doses of curcumin. Colonies were scored after 10-14 days. All experiments were repeated at least three times.” in Materials and Methods to describe our experiments more clearly.

6-Results

It is recommended to delete any numbers and percentages in the text of the results that is shown in figures.

6. Reply: We deleted all numbers and percentages in the text of the results that is shown in figures.

7-Discussion

comment: The manuscript does not adequately address the study’s limitations, such as the relevance of in vitro findings to clinical settings or the potential off-target effects of curcumin. A lack of critical reflection can suggest a biased interpretation of the results.

7. Reply: We added “The limitations are objective, such as…” and “relevance of in vitro findings to the clinical setting needs to be further explored”. At the end of Discussion, we have already referred to “bioavailability of curcumin and off-target effects ”.

8-Conclusion:

There is no clear conclusion section. Please add it

8. Reply: We add “Conclusion: Curcumin exerts anti-neoplastic effects by inducing oxidative stress, DNA damage and mitochondrial dysfunction in ovarian cancer cells, offered novel approach to treating ovarian cancer.”

We are grateful for the invaluable feedback provided by the reviewers. Professional advice and scholarly opinions are very important for our manuscript and further research.

With kind regards,

Weipei Zhu, Ph.D.
